# Towards Decoding Hepatotoxicity of Approved Drugs through Navigation of Multiverse and Consensus Chemical Spaces

**DOI:** 10.3390/biom13010176

**Published:** 2023-01-14

**Authors:** Edgar López-López, José L. Medina-Franco

**Affiliations:** 1 DIFACQUIM Research Group, Department of Pharmacy, School of Chemistry, National Autonomous University of Mexico, Mexico City 04510, Mexico; 2 Department of Pharmacology, Center for Research and Advanced Studies of the National Polytechnic Institute (CINVESTAV), Mexico City 07360, Mexico

**Keywords:** clustering, chemoinformatics, consensus chemical space, data fusion, drug design, drug-induced liver injury, multi-objective optimization, unsupervised learning

## Abstract

Drug-induced liver injury (DILI) is the principal reason for failure in developing drug candidates. It is the most common reason to withdraw from the market after a drug has been approved for clinical use. In this context, data from animal models, liver function tests, and chemical properties could complement each other to understand DILI events better and prevent them. Since the chemical space concept improves decision-making drug design related to the prediction of structure–property relationships, side effects, and polypharmacology drug activity (uniquely mentioning the most recent advances), it is an attractive approach to combining different phenomena influencing DILI events (e.g., individual “chemical spaces”) and exploring all events simultaneously in an integrated analysis of the DILI-relevant chemical space. However, currently, no systematic methods allow the fusion of a collection of different chemical spaces to collect different types of data on a unique chemical space representation, namely “consensus chemical space.” This study is the first report that implements data fusion to consider different criteria simultaneously to facilitate the analysis of DILI-related events. In particular, the study highlights the importance of analyzing together in vitro and chemical data (e.g., topology, bond order, atom types, presence of rings, ring sizes, and aromaticity of compounds encoded on RDKit fingerprints). These properties could be aimed at improving the understanding of DILI events.

## 1. Introduction

Drug-induced liver injury (DILI) is one of the most frequent reasons to stop the drug candidate optimization process (around 67% of these optimizations have been stopped for this issue), and it is the most common feature related to post-marketing withdrawals [1]. For this reason, a current challenge is to enhance the understanding of DILI events. In this context, the current non-multidisciplinary approaches to studying hepatotoxic activity have not been exploiting and combining the large diversity of information (in silico, in vitro, in vivo, and clinical data) available to study this endpoint [2,3].

Recent studies have demonstrated that combining different data types increased the description of DILI events. For example, He et al. demonstrated that the combination of physicochemical and topological descriptors improved the accuracy of predictive DILI models [4]. Thakkar et al. remarked that the compounds associated with DILI events could be classified using mainly anatomical (e.g., drugs used against the nervous system, anti-infectives for systemic use, antineoplastic immunomodulating agents, alimentary tract, and metabolism agents) and therapeutical features (e.g., drugs that act as antidepressants, anti-inflammatory, antirheumatic, and antiviral products) [5]. Furthermore, a recent review by Vall et al. described the potential of artificial intelligence (AI) methods to predict liver injuries, emphasizing that the combination of chemical structures, gene expression, in vitro (e.g., hepatic cytochrome inhibition), in vivo, and imaging assays could be used to decode the side effects of drugs [6]. These recent findings encourage the development of novel methodologies to study a large diversity of data to predict DILI events. The next logical question is, what kind of data and what type of data combinations could help to improve the description of DILI-associated compounds?

In drug design and development, chemical space visualization methods are resources in data mining and information extraction from constantly increasing datasets. Indeed, chemical space visualization is an approach for rationalizing and interpreting experimental and calculated data [7]. Chemical space concept is defined as “an M-dimensional cartesian space in which compounds are located by a set of M physicochemical and/or chemoinformatic descriptors” [8]. Thus, chemical space allows the simultaneous study of different data types, such as structural, chemical, physicochemical, biological, clinical, and/or post-market data, to name a few examples. Since the chemical space depends directly on the descriptors used to define the M-dimensional cartesian space, it is important to mention that it is possible for the coexistence of parallel (or alternative) chemical spaces for the same set of molecules, namely, a multiverse chemical space. In addition, it is possible to combine the alternative chemical spaces to create a single “consensus” chemical space [9]. The chemical space application has demonstrated improvement in drug design, making decisions related to the prediction of structure–properties relationships (SPR), side effects, and polypharmacology drug activity, to mention a few of the most recent advances [10].

In this regard, data fusion methods allow putting multiple data observations or calculations (descriptors) together to increase the consistency and confidence of the information derived from the data [11]. Data fusion was developed initially to improve similarity searching. Data fusion has demonstrated its utility to increase the description of drug design models against different endpoints (e.g., properties, bioactivity, biological pathways, -omics relationships, etc.) from a large data diversity such as structural, physicochemical, spectrometry, bioactivity, transcriptomic, imaging, histological data, etc. [12,13,14,15,16].

The present work aims to improve understanding of DILI events through a novel integration of data fusion concepts using chemical, physicochemical, and biological data, to construct consensus chemical spaces and chemical multiverses.

## 2. Methodology

### 2.1. Dataset Construction and Curation

The dataset was constructed considering data deposited on two major public databases (DrugBank [17] and ChEMBL v.30 [18]) and bibliographic data collected by X. Liu et al. [19] and S. Thakkar et al. [5]. The construction of the dataset used in this work is described as follows:

Liu et al. [19] and Thakkar et al. [5] classified a total of 2309 approved drugs for clinical use according to the reported clinical data that associate each compound with any DILI event. For example, if each compound has been associated (bibliographically) with: fatal hepatic adverse drug reactions, liver failure, liver transplantation, jaundice, bilirubin, liver enzyme increase, hepatomegaly, hepatitis, and/or hepatotoxicity. For this study, compounds associated with almost one of these clinical side effects was considered as “associated with DILI events”. Only 186 (~8%) of the approved drugs were associated with DILI events according with this proposed classification based on clinical data [5,19].

From ChEMBL v.30, a total of 190,068 compounds were retrieved considering the following criteria: molecules tested against the hepatic cell lines HepG2 and Huh7 (ChEMBL ID: 3307718 and 3307515, respectively) and/or the clinically important cytochromes CYP1A2, CYP2A6, CYP2C9, CYP2D6, and CYP3A4 (ChEMBL ID: CHEMBL3356, CHEMBL5282, CHEMBL3397, CHEMBL289, CHEMBL340, respectively).

The approved drugs associated with DILI events and the dataset with cell-based and cytochrome activity data from ChEMBL were merged based on their canonical SMILES. Only 471 compounds (~20% of 2309 approved drugs) are associated with cell-hepatotoxicity activity (HepG2 and/or Huh7) and/or cytochrome inhibition (CYP1A2, CYP2A6, CYP2C9, CYP2D6, and/or CYP3A4). The KNIME software v. 4.7.0 [20] was used to assemble, merge, and curate the datasets. The KNIME workflows are available in the Supplementary Material section (file Multiverse_DataFusion_tSNE.knwf and Multiverse_DataFusion_PCA.knwf).

### 2.2. Descriptor Calculation

Based on the published findings that suggest that the combination of chemical, physicochemical, and structural/topological descriptors improves the classification of DILI-related compounds [4,6], these types of descriptors were calculated in this work.

To describe the chemical and physicochemical context of the dataset, DataWarrior v. 5.5.0 software [21] was used to calculate the number of H-donor bonds, number of H-acceptor bonds, number of rotatable bonds, molecular weight, cLogP, and topological surface area (TPSA) for each compound on the dataset. Additionally, three types of structural/topological descriptors, e.g., Molecular ACCes System (MACCS—166 bits) Keys, RDKit (2048 bits), and ECFP4 (1024 bits) fingerprints were computed using the RDKit [22] module implemented by Python programming language.

### 2.3. Chemical Space Construction

From the dataset with 471 compounds associated with DILI events (available in the Supplementary Material: “DB_ConsensusChemSpace_DILI.csv”), hepatotoxicity cell activity and cytochrome inhibition data were analyzed in their different chemical space representations based on chemical, physicochemical, structural, and in vitro (bioactivity) profile: cytochrome and hepatotoxic cell activity. The implementation of different chemical representations to analyze chemical spaces has been recently termed multiverse chemical space analysis [9].

Before combining all bi-dimensional representations of chemical spaces, each representation was constructed using KNIME software v. 4.3.4 and the module “t-SNE” which is widely used to reduce high-dimensional data to two dimensions [23]. In t-SNE, the parameters were: 1000 iterations, 0.5 theta value, and 30 perplexity values to generate t-SNE 1 and t-SNE 2 coordinates (see file “Multiverse_DataFusion_tSNE.knwf” in the Supplementary Material section).

### 2.4. Assignment of Weights to Each Chemical Space

Before data fusion, it is important to establish the relative importance (weights) of each variable (chemical space coordinates, i.e., t-SNE coordinates) to describe the studied data (chemical structures associated with DILI reports). For this reason, we propose a simple metric, quadrant weight (QW)—Equation (1), that allows uncovering specific regions on the chemical spaces (2D plot coordinates) that are enriched with compounds associated with DILI events:

(1) QW=(−(A∗100)/n)+(NA∗100/n)/2

where “*A*” and “*NA*” represent the number of compounds associated or not with DILI events in a specific quadrant of the chemical space plot, respectively; “*n*” is the total number of compounds contained in the dataset. A positive QW value suggests that a region of the chemical space (2D plot coordinates) is enriched with positive DILI compounds (hepatotoxic). In contrast, negative QW values suggest that a region of the chemical space is enriched with negative DILI compounds (non-hepatotoxic).

**Figure 1:**
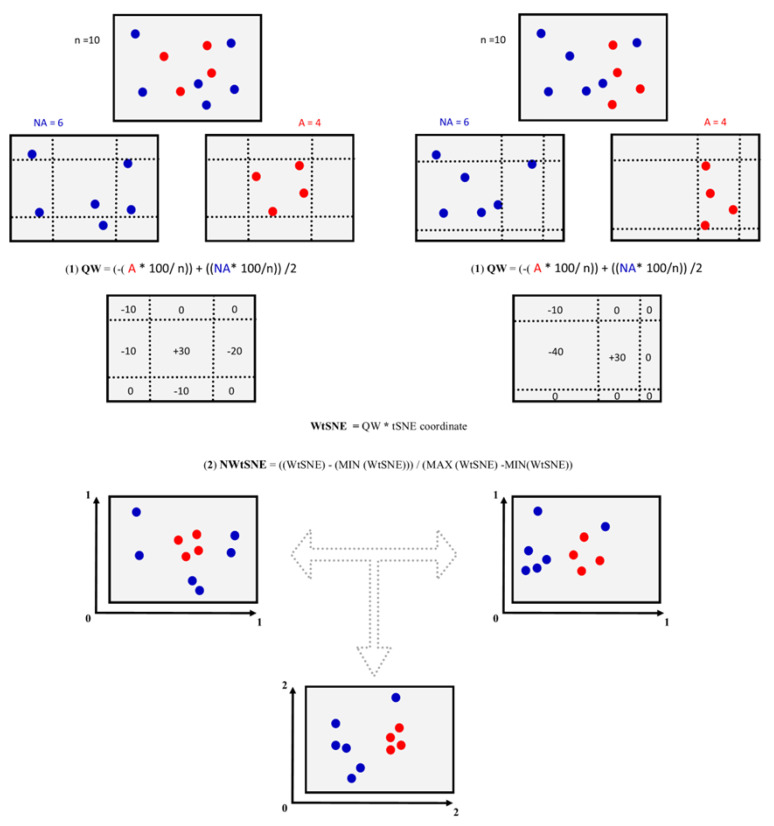
Schematic overview of chemical space construction, assignment of weights to each chemical space, and data fusion protocols implemented in this work.

For this work, we define nine regions of each chemical space representation using the minimum and maximum values of the t-SNE coordinates that contain positive DILI compounds (this step is schematically explained in Figure 1). The criteria to delimit each region are available in the Supplementary material (MetricOfDataFusion.xlsx). Finally, each weight peer quadrant was multiplied by the coordinate (t-SNE 1 or 2) of each compound contained in each chemical space representation.

### 2.5. Data Fusion

Normalized value of weighted t-SNE coordinate (NWtSNE) was calculated to directly compare the representation of the chemical spaces, i.e., based on in vitro data, chemical and physicochemical properties, and fingerprints. Each of the two-dimensional coordinates, t-SNE 1 and t-SNE 2, were calculated using Equation (2):

(2) NWtSNE=((WtSNE)−(MIN(WtSNE)))/(MAX(WtSNE)−MIN(WtSNE))

where “*WtSNE*” is the weighted *t-SNE* coordinate, and “*MIN*” and “*MAX*” are the minimum and maximum *WtSNE* values, respectively.

Finally, the consensus t-SNE coordinates were generated by summing the normalized coordinates of each chemical space representation of each compound. The automatic workflow of this method was implemented in KNIME and it is available in the Supplementary Material (Multiverse_DataFusion_tSNE.knwf). The interactive visualizations of the chemical spaces were generated with DataWarrior software v.5.5.0., and are available in the Supplementary Material (DB_ConsensusChemSpace_DILI.dwar) [21,24]. Figure 1 illustrates graphically an overview of the methodology used in this work: chemical space construction, assignment of weights to each chemical space, and data fusion protocol.

A strategy to evaluate if the clustering of associated and non-associated DILI compounds is efficient is calculating the distance between each compound in each chemical space representation. Namely, the shortest distances between DILI-associated compounds indicate that the clustering method is more efficient. The largest distance in the clustering between DILI-associated compounds indicates that the method is not capable of clustering them. To this end, the Euclidean and Manhattan distances were calculated by each pair of compounds on the dataset [25]. The distances were calculated using the “distance matrix calculate” node in KNIME. The protocol is available in the Supplementary Material (Multiverse_DataFusion_tSNE.knwf). The mean distance between associated (or non-associated) DILI compounds and their standard deviation was calculated and plotted.

## 3. Results

**Figure 2:**
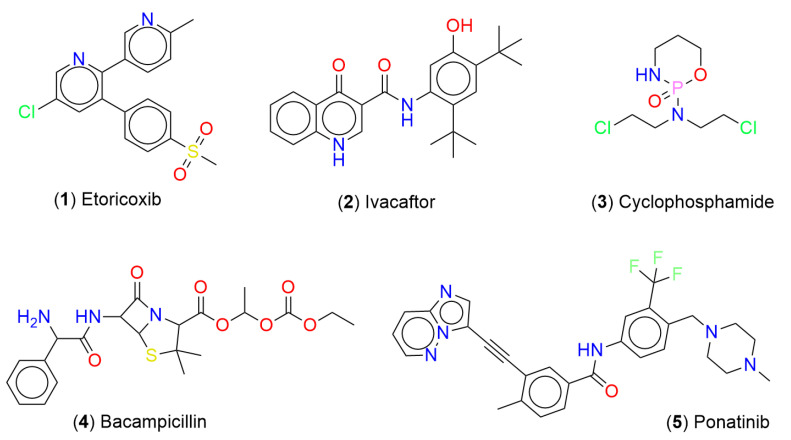
Chemical structures of representative compounds associated with DILI events.

In this section, we discuss the chemical multiverse of compounds associated with DILI reports, and a methodology to integrate chemical space data. Figure 2 shows the chemical structures of representative compounds associated with DILI events. Interestingly, these compounds exhibit a notable structural diversity with different chemical scaffolds, and present different types of atoms (e.g., O, N, S, Cl, F, P, etc.) that confer different kinds of properties.

**Figure 3:**
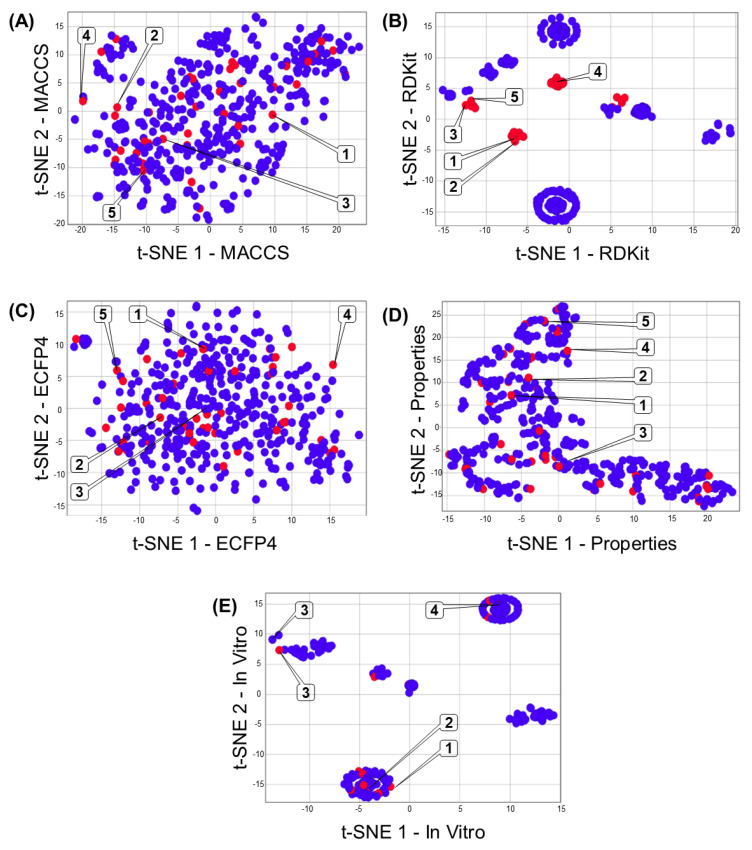
Representation of the multiverse chemical space of 471 compounds associated with DILI events. Each chemical space visualization was constructed by dimensional reduction (t-SNE coordinates) of fingerprints (**A**) MACCS keys, (**B**) RDKit, (**C**) ECFP4, (**D**) chemical and physicochemical properties, and (**E**) in vitro data. Each data point in the graph represents a chemical structure, and the color of these points indicates if the chemical structure has been associated (red) or not (blue) with DILI events. Representative compounds are labeled with the compound numbers as in Figure 1.

Figure 3A–E shows the multiverse chemical space (i.e., different chemical space representations to the same dataset) of 471 compounds associated with DILI reports. Each chemical space representation illustrates structural (e.g., MACCS keys), topological (e.g., RDKit, and ECFP4), chemical and physicochemical (e.g., drug-like properties), or in vitro data of this dataset. The data points colored in red represent compounds associated with DILI events (i.e., compounds associated with hepatotoxic signatures), in contrast with the compounds represented with data points in blue (that have not been related to DILI issues). Figure 3 illustrates an overview of the impact of each kind of descriptor on the clustering of compounds associated with DILI events. For example, the poor clustering generated by data from bidimensional structural descriptors (MACCS fingerprint—Figure 3A) suggests that this information is not enough to cluster the compounds according to their DILI events. In contrast, topological (tridimensional) descriptors (like RDKit) offer a better clustering of compounds associated with DILI events (red dots). Interestingly, the poor clustering based on drug-like properties (Figure 3D) and in vitro data (Figure 3E) suggests that these features (independently) do not guarantee the correct description of DILI events.

**Figure 4:**
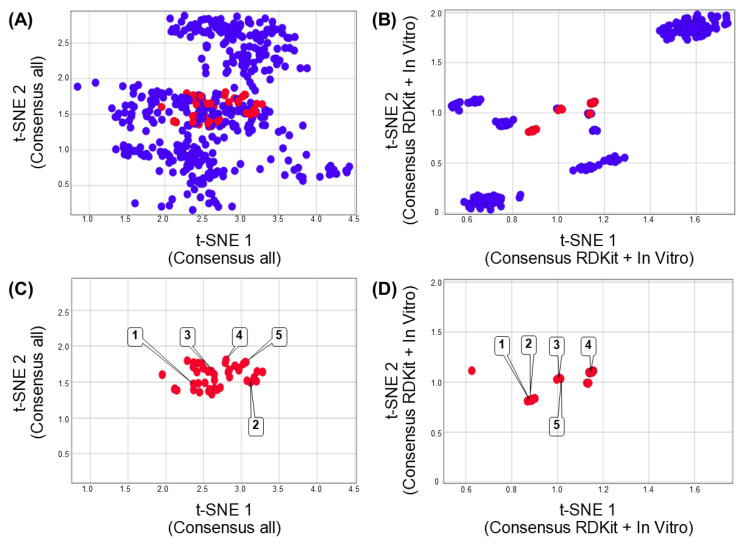
Consensus chemical space of 471 compounds associated with DILI reports. Each chemical space was constructed using the assignment and normalization of the weights by each region of the chemical space. (**A**) Consensus chemical space representation of reduced dimensions generated from fingerprints, chemical/physicochemical properties, and in vitro data related to each compound associated with DILI reports. (**B**) Consensus chemical space representation using the reduced dimensions generated from RDKit fingerprint and in vitro data. (**C**,**D**) Consensus chemical space representations showing only compounds associated with DILI events. Each point in the chemical spaces represents a chemical structure. Data points are colored by if the chemical structure has been associated with DILI events (red) or not (blue). Representative compounds are labeled with the compound numbers as in Figure 1.

Figure 4 shows the consensus chemical space representation. This new chemical space representation improves the visual identification of positive DILI compounds (red data points). Each region of each consensus chemical space representation is constructed, as per Equations 1 and 2, to improve the separation of the positive and negative DILI compound cases. Figure 4A shows the new t-SNE coordinates generated from the fusion of multiverse chemical space data (e.g., structural, topological, chemical, physicochemical, and in vitro data). Figure 4B shows the new coordinates generated from the fusion of structural (RDKit fingerprint) and in vitro data.

It is remarkable the clustering difference observed in the visualization of the chemical spaces generated by only one type of data (Figure 3) as compared to the combined data (Figure 4). Interestingly, the fusion of redundancy data (e.g., using different fingerprints to represent the same molecule, Figure 4A) could not contribute to improving the clustering of DILI compounds.

**Figure 5:**
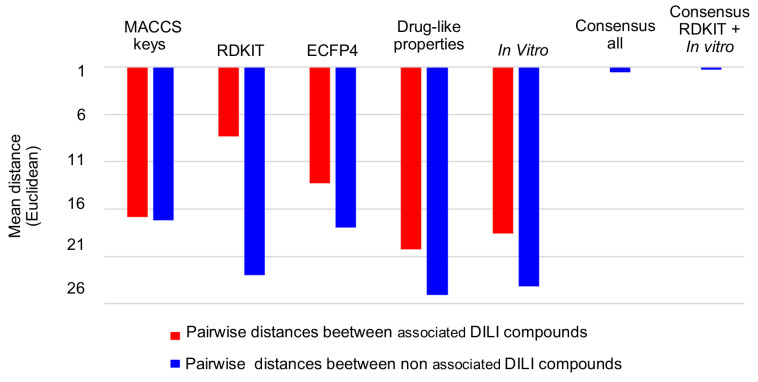
Pairwise Euclidean distances of positive and negative DILI compounds in chemical space representations obtained with different descriptors. The 2D plots of the chemical space visualizations are in Figure 2 and Figure 3.

To remark on the improved clustering of the combined descriptors, the mean pairwise distance of associated (red) and non-associated (blue) compounds with DILI events generated by each chemical space representation was calculated using Euclidean and Manhattan distances (see file “Ditances_ChemSpaces.xlsx” in the supplementary material section): Remarkably, Euclidean distance allows the reduction of the distance of compounds associated with DILI events (red), especially using properties and in vitro data, in contrast with Manhattan distance. Figure 5 indicates that the use of a single data type generates a higher average pairwise distance (low clustering efficiency) of positive DILI compounds (from 8.3 to 20.3), and paired negative DILI compounds (from 17.1 to 25.1). This is in contrast with the consensus chemical space representation (fused data) that exhibits lower mean pairwise distance (high clustering efficiency) between positive DILI compounds (from 0.24 to 0.28) and negative DILI compounds (from 1.26 to 1.56).

Interestingly, using fused data, the distance between the non-associated DILI compounds continues to be higher than the distance between associated-DILI compounds. This fact suggests that the non-associated DILI compounds exhibit a higher intrinsic chemical diversity.

Each representation offers a unique form to cluster each chemical structure (Figure 3 and Figure 4). However, consensus methods provide a mathematical framework to establish a weight for each region on the different chemical space representations (generating a semi-unsupervised approach to construct enriched chemical space representations, Figure 3). From a pharmacological view, these results remark on the importance of multidisciplinary approaches, using chemical and biological data, to develop methodologies capable of efficiently describing DILI events.

## 4. Discussion

There are multiple representations available to describe compounds and study the structure–property relationships (SPR) of a dataset. The large variety of molecular descriptors is linked to the subjectivity of the “molecular similarity” that is dependent on the molecular representation [26]. Namely, the similarity of a pair of compounds depends on the features used to compare them. In fact, a pair of compounds could be considered similar if we use structural descriptors, but this does not guarantee that both compounds have similar in vitro activity [27]. For this reason, it is crucial to evaluate the similarity of the compounds and, in general, the SPR of datasets using different descriptors and similarity metrics. The combined analysis of alternative representations (also known as data fusion) could reduce the information gap between the chemical structures vital in drug development and biological knowledge. However, one of the most important issues in data fusion is assigning adequate weights to each variable that is being combined (e.g., dimensions that define the compound’s chemical space) because different mathematical approximations could be used to generate them [28]. In fact, there is no unique and “best” manner to generate consensus chemical spaces. Namely, it is necessary to adapt the data fusion approach to consider each dataset. This important point could lead to feature selection for prospective studies, generating a good starting point for exploring large datasets.

There is a crescent interest in developing protocols capable of predicting DILI events. However, these side effects are complicated to predict because they are associated with (parallel) multiple pharmacological and toxicological events and become a typical problem to address with multiple-parameter optimization. For example, existing reports demonstrate the relationships between chemical structures and physicochemical properties with DILI events, but at the same time, other authors show that ADME properties, cell-based data, and other in vitro assays lead to the identification/prediction of DILI events. Namely, the DILI events are a complex case study that requires using all available data to rationalize (almost in part) and predict their occurrence during pre-clinical and clinical interventions. Fortunately, the current multi-objective optimization methods could help address this issue briefly [29].

Consensus chemical spaces are an approach to fuse and use different kinds of data (e.g., descriptors that define the multidimensional vector space) to improve predicting a specific, desired property. To this end, the main challenge is to choose from the several methods available to combine high dimensionality of data using a robust mathematical scheme.

Additionally, and as happens in any other predictive methodology, another major issue to address is the limited access to data [30], considering that several results that are regarded as of “no interest” for a particular study (at some point in time) are rarely published. This fact creates a crescent gap in the available information related to compounds associated with poor activity or side effects like DILI events. For example, as was mentioned in Section 2.1 of this manuscript, only 471 compounds have associated with “complete” information related to their chemical, physicochemical, and biological data, namely, not all compounds have in vitro data (cytochrome and cell-based inhibition data) to compare. In fact, this is the main limitation of “data fusion” methodologies.

For prospective studies, it will be necessary to assess multiple methods to fuse data [31] and use other high-dimensional reduction methods [32]. For example, in addition to using tSNE methods (non-linear reductional dimension method) to represent the chemical space of DILI compounds, it is possible to adapt other methods such as principal component analysis (PCA—linear reductional dimension method, see supplementary material: Multitiverse_DataFusion_PCA.knwf) to describe the multiverse and consensus chemical spaces of DILI compounds. However, the implementation of the PCA analysis to the DILI dataset does not allow the clustering differentiation between associated and non-associated DILI compounds. This could be explained by the low correlation between each descriptor (i.e., fingerprints, properties, and in vitro data). For the current dataset, the chemical space representation of DILI compounds obtained from PCA does not show an improvement using data fusion. For this reason, we highlight the importance of assessing different reductional dimension methods according to specific datasets.

The DILI understanding is relevant to elucidating molecular mechanisms, identifying novel biomarkers, and preventing drug side effects prior to pre-clinical and clinical interventions. The multiverse chemical space and the consensus chemical space representations (using fused data) enrich the information that could generate useful knowledge. For example, the drug design methods based on fused data could improve the next generation of toxicological and post-marketing decision-making approaches.

The results illustrated in Figure 4 show that the RDKit fingerprint allows more efficient clustering in contrast with other types of fingerprints and descriptors explored in this work. For example, ECFP4 is a circular fingerprint meaning that each atom on each molecule could be described by the topology and bond order, considering only four atoms to distance. In contrast, the RDKit fingerprint also considers atom types, the presence/absence of rings, and aromatic systems. This observation highlights the importance of the intrinsic descriptor encoded by the RDKit fingerprint (e.g., topology, bond order, atom types, presence of rings, ring sizes, and aromaticity of each compound) that could be used to improve the understanding of DILI events.

**Figure 6:**
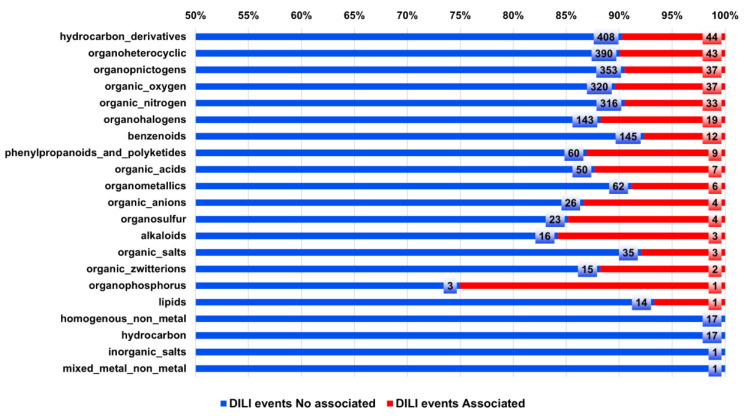
Types of compounds and their association with DILI events. A total of 471 compounds associated with DILI reports were classified [33] according to their chemical taxonomy, and each chemical taxonomy was associated with the number of cases associated (red) and no associated (blue) with DILI events.

Figure 6 shows a classification of the 471 compounds associated with DILI according to the type of chemical taxonomy. The analysis shows that major types of compounds exhibit around 10% of chemical structures associated with DILI events. However, organohalogens, phenylpropanoids, polyketides, organic acids, organosulfur, alkaloids, and organophosphorus compounds exhibit a rate higher than 10% of associated DILI compounds.

Additionally, the most frequent compounds associated with DILI events contain complex ring systems, specific functional groups, and atoms (e.g., double bonds, carboxylic acids, ketones, halogens, sulfur, phosphorus) that per se have been associated with hepatic injuries [34,35,36,37,38] (see exemplary chemical structures in Figure 2). From a chemical perspective, these observations could lead to the early identification of compounds potentially associated with DILI events.

From a pharmacological perspective, we remark on the importance of incorporating data that predict the hepatic and microbiota biotransformation [39,40] of xenobiotics to increase the early identification of potential associated DILI compounds. Acetaminophen provides a typical example of the importance of studying biotransformation. This drug is not hepatotoxic but its metabolites generate fulminant liver injuries [41,42].

Finally, we need to clarify that the present methodology represents a new alternative to preparing and filtering useful data to develop predictive models (e.g., machine learning models). However, there are multiple possibilities to fuse data, different kinds of criteria to select the input information, and a large list of predictive models to obtain output data. For all these reasons, this study does not pretend to resolve the DILI prediction problem, but aims to introduce a new approach to integrate different criteria towards decoding hepatotoxicity of approved drugs (as mentioned in the title of this work).

## 5. Conclusions

DILI is the principal reason for failure in developing drug candidates. It is the most common reason to withdraw from the market after a drug has been approved for clinical use. However, the current approaches to predicting DILI have not allowed a complete understanding of chemical and biological alerts to identify early compounds associated with DILI events.

Drug design methodologies based on fused data could be the next generation of tools used in rational design, especially to decode complex pharmacological issues such as DILI events. Here, we introduce a combined analysis of DILI-related events using the concept of consensus chemical space and the chemical multiverse, using chemical, physicochemical, structural, biochemical, and biological data to improve the understanding of DILI events. Our results, which suggest that the combination of chemical structural and biological data improves the clustering of associated DILI compounds, pave the way to new opportunities to develop predictive models (like machine and deep learning models) capable of predicting DILI events in an early stage of the drug development process. It was also concluded that organohalogens, phenylpropanoids, polyketides, organic acids, organosulfur, alkaloids, and organophosphorus compounds are associated with a higher rate of DILI events. For this reason, we suggest more exhaustive preliminary studies for these types of compounds with the aim of reducing the cases associated with DILI events.

## Data Availability

The original contributions presented in the study are included in the article and Supplementary Material. Further inquiries can be directed to the corresponding authors.

## Supplementary Materials

The Supplementary Material for this article can be found online at: https://doi.org/10.5281/zenodo.7526385.
